# Walking activity and depressive symptoms among Mexican American older adults aged 80 years and older

**DOI:** 10.1093/geroni/igaf122.2719

**Published:** 2025-12-31

**Authors:** Kevin Chen, Soham AlSnih

**Affiliations:** UTMB John Sealy School of Medicine, Houston, Texas, United States; The University of Texas Medical Branch at Galveston, Texas, Galveston, Texas, United States

## Abstract

Physical activity is known to be protective for the development of depressive symptoms in older adults. We examined the relationship between walking activity and depressive symptoms among Mexican American older adults without a history of depressive symptoms at baseline over a 9-year period. Participants (N = 836) were Mexican American aged 80 years and older living in the southwestern United States (Arizona, California, Colorado, New Mexico, and Texas) from the Hispanic Established Population for the Epidemiologic Study of the Elderly (2007/05-2016). Measures included socio-demographics and health characteristics. Depressive symptoms were assessed with the Center for Epidemiologic Studies Depression Scale (CES-D ≥ 16). At baseline, participants were grouped into non-walkers (n = 535), walked < 150 minute/week (n = 123), and walked 150 minutes/week or more (n = 178). Generalized Estimating Equation models estimated the odds ratio (OR) and 95% Confidence Interval (CI) of depressive symptoms as a function of walking activity status controlling for all covariates. We found that those who walked ≥150 minutes/week had lower odds (OR = 0.53, 95% CI = 0.30-0.93) of developing depressive symptoms over time compared to non-walkers after controlling for all covariates. There was a non-significant relationship between walking < 150 minutes/week and depressive symptoms (OR = 0.81, 095% CI = 0.51-1.31) over time compared to non-walkers after controlling for all covariates. In conclusion, Mexican American older adults who engaged in any walking activity were at reduced risk of developing depressive symptoms. Preventing depression in this growing population may improve their quality of life and reduce their burden of care.

